# Frequency and predictors of premature work loss in primary care consulters for osteoarthritis: prospective cohort study

**DOI:** 10.1093/rheumatology/ket336

**Published:** 2013-10-17

**Authors:** Ross Wilkie, Chris Phillipson, Elaine Hay, Glenn Pransky

**Affiliations:** ^1^Arthritis Research UK Primary Care Centre, Primary Care Sciences, Keele University, Keele, Staffordshire, ^2^School of Social Sciences, University of Manchester, Manchester, UK and ^3^Center for Disability Research, Liberty Mutual Research Institute, Hopkinton, MA, USA.

**Keywords:** osteoarthritis, work disability, epidemiology, primary health care, prospective study

## Abstract

**Objective.** The objective of this study was to describe the extent of premature work loss (PWL) in OA consulters across a 6-year observation period, and associated factors.

**Methods.** We conducted a population-based prospective cohort study set in primary care. Participants were 1098 adults age 50 years to statutory retirement age at baseline, who completed questionnaires at baseline, 3- and 6-year follow-ups. OA was defined by consultation to primary care (Read code N05) during the study period. PWL was defined as retirement prior to state retirement age (65 years for men, 60 years for women), off work due to health or unemployment. The frequency of PWL was calculated overall and stratified by consultation for OA. Bivariate and multivariate logistic regression was used to investigate the predictors of PWL in consulters for OA.

**Results.** Over the 6-year study period, one in four consulters for OA left the workplace prematurely. Predictors included being male, pain interference with function and lower co-worker support, but not the extent of arthritis, co-morbidity, obesity or psychological or other job factors.

**Conclusion.** PWL in persons consulting primary care general practitioners with OA is common. Those at risk could be identified by brief questions about pain interference with function and workplace support. These results suggest that early identification, treatment strategies focusing on maintaining function and maximizing workplace support should be investigated for their potential to prevent PWL. Good communication with employers may help to improve support for workers with OA.



## Introduction

OA is the most common joint condition in adults and globally is the fastest increasing major heath condition [1]. It is recognized as one of the leading and rapidly growing causes of disability [2]. Loss of work participation is one form of disability and will become more important as adults work to older ages due to rising state pension age and have greater financial needs resulting from inadequate retirement resources [3]. The health and economic benefits of staying at work are becoming apparent, thus maintaining employment of persons with OA is an international priority [4].

OA is a common reason for primary care consultation (1 in 20 consultations in adults age 45–65 is for OA [5]), yet we do not know how many of these patients are at risk of premature work loss (PWL) and why. Based on a representative cohort of primary care consulters, this study reports the frequency of and the factors that predict PWL across a 6-year observation period in patients with OA.

## Methods

### Study population

The North Staffordshire OA project is a population-based prospective cohort study. All individuals age ≥50 years (*n* = 19 818) registered with six general practices were mailed a baseline questionnaire in 2002 that collected data on health, socio-demographic factors and pain, with follow-up questionnaires 3 and 6 years later. Reminders were sent to non-responders 2 and 4 weeks after the initial mailing. The North Staffordshire Local Research Ethics Committee approved this study; all participants gave written informed consent to participate.

**Fig. 1:**
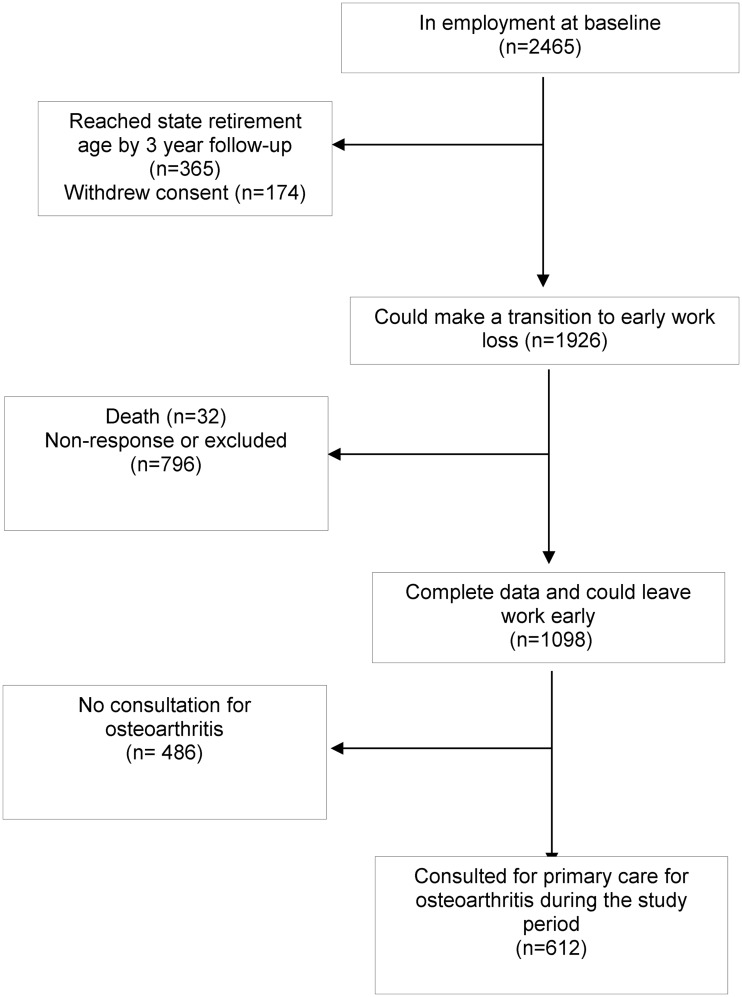
Flow diagram of participants for the longitudinal analysis.

Analyses included those who consented to medical records review, were of working age at baseline (<60 years for women and <65 years for men), could move from employment to PWL during the 6-year follow-up period and had complete data. Of 2465 potential participants employed at baseline, exclusions included 365 who reached retirement age before the 3-year follow-up, 174 who withdrew, 32 who died and 796 who did not respond at 3 or 6 years, leaving complete data for 1098 participants (adjusted response rate 58.0%) (Fig. 1). Compared with those subjects who withdrew, died, did not respond or had incomplete data (*n* = 1002), included participants were on average slightly younger, had higher occupational status, were more educated and had more multimorbidity and consultations for OA.

### Identification of OA

General practitioners in the study used the Read system to code all reasons for clinical encounters in primary care consultations [6]. Morbidity data (i.e. symptoms and diseases) in this system are grouped under 19 main Read chapters. Data collected at the second hierarchical level or above were used to identify diagnostic groups, and these were aggregated starting 18 months before the baseline questionnaire was administered and continued through the time of the final follow-up questionnaire. Individuals were defined as having OA if they had at least one consultation during this period for OA based on Read codes (N05 category) for primary care consultations [6].

### Outcome measure

PWL was defined as moving from employment to retirement prior to state retirement age or transition from employment to being off work due to health or unemployment between baseline and 3 years or between 3 and 6 years using the participant’s self-report of employment status [employed (including self-employed), off work due to illness, unemployed, homemaker, retired].

### Independent factors

All independent factors were measured at baseline except for workplace factors, measured at the 6-year follow-up. Pain interference was measured using the 12-item Short Form (SF-12) question, ‘During the past 4 weeks, how much did pain interfere with your normal work (work/housework)?’ [7] and was classified as high (moderately, quite a bit or extremely) or low (not at all or a little bit). Single dichotomous items measured the presence of joint pain in the hands, hips, knees and feet, the areas where OA is most common [2]. A ‘number of joint pains’ was calculated by counting the number of different anatomical sites involved (0–4) with no distinction between unilateral and bilateral pain (e.g. unilateral and bilateral knee pain = 1). The extent of musculoskeletal pain over the previous 4 weeks was measured by responders shading painful areas (0–44) on a full-body diagram (front and back views). Scores of the number of shaded areas were categorized into three groups (0, 1–5 areas, 6–44 areas).

Comorbidity was identified using Read diagnostic codes from primary care consultations during 18 months before the baseline questionnaire. Multimorbidity was defined as four or more comorbidities (different major diagnostic groups) [8]. Anxiety and depression during the previous week were measured using the Hospital Anxiety and Depression Scale; raw scores categorized individuals as non-cases (0–7) or possible/probable cases (8–21) [9]. Self-reported height and weight were categorized into standard BMI groups: (i) normal weight (BMI 20–24.9 kg/m^2^), (ii) underweight (BMI <20 kg/m^2^), (iii) overweight (BMI 25–29.9 kg/m^2^) and (iv) obesity (BMI ≥30 kg/m^2^). Perceived control of health was measured using a single item (‘There is a lot I can do to control my health: yes/no?’). Demographic and socio-economic details included age, gender, educational attainment [finished education on leaving grade school, went on to further education (college or university)] and occupational class (managerial and professional, intermediate, routine).

Single items on the 6-year follow-up questionnaire measured workplace characteristics. Items for those who were employed, out of work and retired were combined to provide an overall measure of each workplace factor (i.e. physical demands and co-worker support). To measure physically demanding employment the following items were combined: for those in employment, ‘my work is physically demanding: agree/disagree’; for those out of work or retired, ‘would fewer physical demands have prevented movement out of work/facilitate return to work: yes/no’. To measure co-worker support: for those in employment, ‘my work colleagues are supportive: agree/disagree’; for those not in work or retired, ‘would more support from colleagues or fewer demands have prevented movement out of work/facilitated return to work: yes/no’.

### Statistical analysis

The frequency of PWL was calculated overall and stratified by consultation for OA (yes/no); frequencies were compared using the percentage difference with 95% CIs. Differences between the categories of work loss were compared using a chi-squared test.

To investigate the predictors of PWL in consulters for OA, the relationship between each independent factor and PWL, adjusting for potential confounders (age, gender and socio-economic factors), was examined using logistic regression. Factors significantly associated with PWL (*P* < 0.05) were then included in a final multivariate model. Analysis was performed using Stata version 12.

## Results

Of the 1098 participants, 612 [55.7%; mean age 54.6 (s.d. 2.8) years, 48.2% were female] consulted for OA during the study period. Compared with those who did not consult for OA, those who did had lower physical health (SF-12 physical health component score: 46.7 *vs* 50.9, *P* < 0.001) were more likely to be female (48.2% *vs* 39.9%, *P* = 0.01), have multimorbidity (33.5% *vs* 15.6%, *P* < 0.001) and be obese (21.6% *vs* 11.7%, *P* = 0.001), however, there was no difference for age (54.6 *vs* 54.4, *P* = 0.20), mental health (SF-12 mental health component score: 49.7 *vs* 50.9), physically demanding jobs (36.7 *vs* 37.2, *P* = 0.13), low job support (10.3% *vs* 7.6%, *P* = 0.13) or low control of health (7.0% *vs* 4.7%, *P* = 0.87).

Overall, 259 individuals (23.6%) experienced PWL. The estimated frequency of early work loss was not significantly higher in consulters for OA compared with those without OA [24.2% *vs* 22.8%; percentage difference 1.3% (95% CI 3.7, −6.3)]. However, the reason for early work loss differed between the two groups (*P* = 0.03); those who consulted for OA were more likely to be off work due to sickness (33.8% *vs* 19.1%). In those who consulted for OA, PWL was associated with increasing age [odds ratio (OR) 1.12, 95% CI 1.05, 1.20], male gender (adjusted OR 1.95, 95% CI 1.28, 2.97), pain interference (OR 1.58, 95% CI 1.05, 2.36) and low co-worker support (OR 3.26, 95% CI 1.87, 5.68) (Table 1). In the final multivariate model, male gender (OR 1.97, 95% CI 1.28, 3.04), pain interference (OR 1.51, 95% CI 1.00, 2.27) and low co-worker support (OR 3.11, 95% CI 1.78, 5.42) were the three factors independently associated with PWL.

**Table 1:** Associations between health, demographic, socio-economic and environmental factors and PWL in adults with OA (*n* = 612)

		Adjusted OR		Final model OR	
Factors	Number (%) who experience PWL	OR^a^	95% CI	OR^b^	95 %CI
Age				1	
		1.07	0.99, 1.15	1.07	0.99, 1.15
Gender					
Female	49 (16.6)	1		1	
Male	99 (31.2)	1.95	1.28, 2.97	1.97	1.28, 3.04
Educational attainment					
Further	26 (21.7)	1		1	
School only	122 (25.3)	0.80	0.47, 1.35	0.86	0.50, 1.48
Occupational classification					
Managerial/professional	42 (27.3)	1		1	
Intermediate	25 (26.6)	0.89	0.48, 1.63	0.78	0.42, 1.45
Routine	80 (22.2)	0.69	0.43, 1.11	0.65	0.40, 1.06
Pain interference					
None/a little	92 (21.4)	1		1	
Moderate/quite a bit/extremely	56 (31.6)	1.58	1.05, 2.36	1.51	1.00, 2.27
Number of joints with pain					
0	35 (29.4)	1		—	—
1	30 (17.1)	0.52	0.29, 0.91		
2	33 (22.5)	0.67	0.38, 1.18		
3	29 (29.0)	1.06	0.58, 1.93		
4	14 (26.4)	0.95	0.45, 2.00		
Number of pains					
0	41 (30.6)	1		—	—
1–5	38 (19.0)	0.51	0.30, 0.86		
≥6	69 (24.8)	0.74	0.46, 1.18		
Comorbidity					
Low comorbidity (0–3)	99 (24.3)	1		—	—
Multimorbidity (≥4)	49 (23.9)	1.03	0.69, 1.55		
Depression					
Non-case (0–7)	126 (23.8)	1		—	—
Possible/probable case (8–21)	22 (27.9)	1.28	0.73, 2.22		
Anxiety					
Non-case (0–7)	91 (24.7)	1		—	—
Possible/probable case (8–21)	57 (23.7)	0.99	0.67, 1.46		
BMI					
Normal (20–24.9 kg/m^−2^)	48 (24.98)	1	1	—	—
Underweight (<20)	4 (40.0)	2.07	0.53, 8.07		
Overweight (25–29.9 kg/m^2^)	55 (20.9)	0.68	0.43, 1.07		
Obese (>30 kg/m^2^)	40 (30.3)	1.25	0.75, 2.08		
Control of health					
Can control health	135 (23.7)	1		—	—
Can’t control health	13 (30.2)	1.47	0.73, 2.96		
Physically demands of job					
Not physically demanding	104 (26.9)	1		—	—
Physically demanding	44 (19.6)	0.64	0.42, 0.96		
Co-worker support					
Good co-worker support	119 (21.7)	1		1	
Low co-worker support	29 (46.0)	3.26	1.87, 5.68	3.11	1.78, 5.42

OR = 1 for each referent subgroup in multivariate comparisons. ^a^OR adjusted for potential confounders (age, gender and socio-economic status). ^b^OR adjusted for age, gender, socio-economic status and factors significantly associated with PWL after adjustment for potential confounders.

## Discussion

This study is the first to report the rate of PWL in adults with OA. PWL is common (almost one in four with OA leave work prematurely), and because of the frequency of OA there are significant numbers of adults with the condition who are leaving work prematurely. The rate is comparable to that of those who consult for other, often serious, medical conditions in this age group, and highlights the impact of OA, which is often regarded as relatively benign. Notably, the prevalence of PWL at baseline (i.e. the proportion who at baseline had retired prior to state retirement age or were off work due to sickness or unemployment) was significantly greater than in those who had not consulted for OA (35.4% *vs* 26.2%, *P* < 0.001). Men who consult for OA are particularly vulnerable to PWL, as well as those with greater pain interference and low workplace support; several other factors were not significant.

PWL in those with OA is driven by its main characteristic, pain interference (i.e. functional limitation due to pain), which was independently associated with PWL, but not the number of different joints involved, bodily distribution of pain, mood or comorbidities. The severity of OA has previously been linked with lower productivity [10], but this finding reinforces prior observations that functional impact due to pain is the key factor in determining ability to work [11]. Medical approaches to managing OA symptoms are important, but the main challenge in relation to maintaining employment is how to manage functional limitations. Targeting pain per se may not prevent PWL, as suggested by studies in low back pain and other conditions (e.g. [12]). However, targeting the psychological mechanisms perpetuating pain, and maintaining physical capacity, can lead to significant improvements in function, and improving coping mechanisms can lead to improved work outcomes in OA [13, 14].

Lack of support from work colleagues emerged as the other strong predictor of PWL in persons with OA. This finding is consistent with other studies that emphasize the importance of workplace support and accommodations in enabling workers with chronic conditions to avoid work disability altogether [15]. A high level of pain interference may itself reflect inadequate workplace accommodations to the symptoms of OA, a possible consequence of low co-worker support. This increased difficulty on the job may instigate primary care consultation.

Improving workplace support indicates a need for clinicians to communicate with employers. Clinicians must increasingly look at the role of, and interaction with, line managers, return-to-work co-ordinators and human resources in the workplace. Line managers need to consider the symptoms and functional limitations of workers with OA and pain to minimize work loss. Clinicians can advise line managers on the symptoms and management of OA in the workplace, suggesting strategies such as flexible hours, amended duties, job rotation and workplace adaptation [15]. In this regard, clinicians have a high degree of credibility and influence, although this is infrequently exercised to effect positive changes on their patients’ behalf [16]. Other professionals have more training and expertise in this area. Depending on the particular context, available public and private resources and vocational and other services can be engaged by clinicians to provide this expertise and support to prevent PWL. Reducing PWL may depend more on competencies in ergonomic job accommodation, communication and conflict resolution than on direct management and care of OA [17]. Although communication with co-workers and supervisors about arthritis may help to increase support, those with OA may perceive self-disclosure as counterproductive in a relatively unsupportive work environment [18]. However, early recognition and intervention is important—compared with other disabling musculoskeletal conditions, those who finally leave work due to OA may be at highest risk of never returning to the workplace [19].

The strength of this study’s longitudinal design enables prospective identification of factors associated with PWL in a clinically relevant primary care population. Estimates of the relative impact of OA on work have not been available; different populations (e.g. national samples *vs* clinical, self-report pain *vs* diagnosed OA) and outcomes and the lack of a longitudinal approach have obscured the extent of OA’s impact on work and associated factors [20]. The sample is representative of primary care consulters with physician-diagnosed OA. Other studies have been limited to patients from rheumatology practices or rehabilitation clinics, a less representative sample of OA patients (e.g. [18]).

There are limitations to this study. Data on most variables were by self-report, but validated instruments were used to measure anxiety, depression and pain interference. We did not have radiographic or detailed information on the extent of OA, but the intention of the study was to describe a typical, heterogeneous group of patients with OA as seen in primary care practice. Workplace variables were slightly different depending on whether responders were in employment, and were not obtained prospectively. Baseline measurement of predictors may not reflect changes in these factors during follow-up. The heterogeneous outcome variable (PWL defined by early retirement, off work due to health and unemployment) obscures specific reasons for each of these outcomes; ideally each outcome would have been examined separately, but the study did not have an adequate sample size for these separate analyses. Attrition and missing data indicate there may be some bias due to differences in age, gender, socio-economic status and health status between those who dropped out and those included, but this is likely to be minimal, as the differences between the participants and non-participants on key variables were not large. Radiologic diagnostic or specific clinical measurements of OA were not available, but this study was intended to describe the outcomes in a typical group of patients who receive a diagnosis of OA in primary care.

**Rheumatology key messages**

- Premature work loss in primary care consulters for OA is common.
- Management of functional limitations and effective communication with employers can improve work participation for workers with OA*.*

## References

[1] Murray CJ, Vos T, Lozano R (2012). Disability-adjusted life years (DALYs) for 291 diseases and injuries in 21 regions, 1990–2010: a systematic analysis for the global burden of disease study 2010. Lancet.

[2] Arden N, Nevitt MC (2006). Osteoarthritis: epidemiology. Best Pract Res Clin Rheumatol.

[3] Helman R, Copeland C, Van Derhei J (2012). The 2012 Retirement Confidence Survey: job insecurity, debt weigh on retirement confidence, savings. EBRI Issue Brief.

[4] Waddell G, Burton AK (2006). Is work good for your health and well-being?.

[5] Jordan K, Clarke AM, Symmons DP (2007). Measuring disease prevalence: a comparison of musculoskeletal disease using four general practice consultation databases. Br J Gen Pract.

[7] Ware J, Kosinski M, Keller SD (1996). A 12-item Short-Form Health Survey: construction of scales and preliminary tests of reliability and validity. Med Care.

[8] Kadam UT, Croft PR, North Staffordshire GP, Consortium Group (2007). Clinical multimorbidity and physical function in older adults: a record and health status linkage study in general practice. Fam Pract.

[9] Zigmond AS, Snaith RP (1983). The hospital anxiety and depression scale. Acta Psychiatr Scand.

[10] Sadosky AB, Bushmakin AG, Cappelleri JC (2010). Relationship between patient-reported disease severity in osteoarthritis and self-reported pain, function and work productivity. Arthritis Res Ther.

[11] Björk M, Thyberg I, Rikner K (2009). Sick leave before and after diagnosis of rheumatoid arthritis—a report from the Swedish TIRA project. J Rheumatol.

[12] Guzman J, Hayden J, Furlan AD (2007). Key factors in back disability prevention: a consensus panel on their impact and modifiability. Spine.

[13] Linton SJ, Nordin E (2006). A 5-year follow-up evaluation of the health and economic consequences of an early cognitive behavioral intervention for back pain: a randomized, controlled trial. Spine.

[14] Gignac MA (2005). Arthritis and employment: an examination of behavioral coping efforts to manage workplace activity limitations. Arthritis Rheum.

[15] Franche RL, Cullen K, Clarke J (2005). Workplace-based return-to-work interventions: a systematic review of the quantitative literature. J Occup Rehabil.

[16] Welsh VK, Mallen CD, Wynne-Jones G (2012). Exploration of GPs’ views and use of the fit note: a qualitative study in primary care. Br J Gen Pract.

[17] Shaw W, Hong QN, Pransky G (2008). A literature review describing the role of return-to-work coordinators in trial programs and interventions designed to prevent workplace disability. J Occup Rehabil.

[18] Gignac MA, Cao X (2009). “Should I tell my employer and coworkers I have arthritis?” A longitudinal examination of self-disclosure in the work place. Arthritis Rheum.

[19] Gjesdal S, Bratberg E, Maeland JG (2009). Musculoskeletal impairments in the Norwegian working population: the prognostic role of diagnoses and socioeconomic status: a prospective study of sickness absence and transition to disability pension. Spine.

[20] Bieleman HJ, Bierma-Zeinstra SM, Oosterveld FG (2011). The effect of osteoarthritis of the hip or knee on work participation. J Rheumatol.

